# Performance evaluation of a point of care cartridge of the new GEM Premier ChemSTAT analyzer

**DOI:** 10.1016/j.plabm.2022.e00297

**Published:** 2022-07-19

**Authors:** Alba E. García-Fernández, Raquel Barquín, Mariano Martínez, Roser Ferrer, Ernesto Casis, Clarke Xu

**Affiliations:** a Clinical Laboratories, Vall d'Hebron Hospital Campus, Barcelona, Spain; b Werfen, Bedford, MA, USA

**Keywords:** Point-of-care, Chemistry panel, Creatinine, Blood urea nitrogen, Estimated glomerular filtration rate, Acute kidney injury

## Abstract

**Background and aims:**

GEM Premier ChemSTAT is a new point-of-care system providing rapid creatinine, BUN and tCO_2_ measurements together with electrolytes, metabolites, hematocrit, pH and pCO_2_ from a single whole blood specimen in acute care settings such as emergency departments and intensive care units. Accurate measurements of whole blood creatinine can aid in the diagnosis and treatment of renal diseases.

**Materials and methods:**

Heparinized whole blood samples from different clinical locations were evaluated on the GEM Premier ChemSTAT and results compared to plasma from the same samples on the Beckman AU5800 or whole blood on the GEM Premier 4000. Precision studies were conducted with whole blood and quality control material.

**Results:**

ChemSTAT correlated well with plasma samples on the AU5800 (regression slopes (S): 0.957–1.159, correlation coefficients (r)≥0.952) and with whole blood specimens on the GEM Premier 4000 (S: 0.9646–1.124, r ≥ 0.974). The repeatability was 0.1%–3.1% and QC precision were within lab and manufacturers’ specifications.

**Conclusion:**

ChemSTAT demonstrated strong correlation to the comparative methods and excellent precision. Combining with its continuous quality management, ChemSTAT is suitable for acute care settings to provide rapid, reliable results, which could minimize time-to-treatment and improve patient outcome.

## Highlights

• GEM Premier ChemSTAT evaluated against Beckman AU 5800 and GEM Premier 4000.
• A multi-use cartridge-based system measures twelve analytes in whole blood sample.
• Method comparison and precision meet laboratory and manufacturers' specifications.
• Whole blood creatinine assays showed lab quality performance.
• Plasma vs serum bias observed for creatinine and urea on lab analyzer.

## Abbreviations

BUN: blood urea nitrogen
POC: point of care
eGFR: estimated glomerular filtration rate
IDMS: isotope dilution mass spectrometry
MDRD: Modification of Diet in Renal Disease
CKD-EPI: Chronic Kidney Disease Epidemiology Collaboration
NKDEP: National Kidney Disease Education Program
MDL: medical decision level

## 1 Introduction

A whole blood point-of-care system that can perform a quick metabolic chemistry panel consisting of sodium (Na^+^), potassium (K^+^), calcium (Ca^++^), chloride (Cl^−^), glucose, creatinine, blood urea nitrogen (BUN), total carbon dioxide (tCO_2_), lactate, pH and pCO_2_ in one whole blood specimen could help patient triage, monitoring and treatment. Literature has provided evidence for the relevance of electrolyte abnormality, acute kidney injury (AKI) and chronic kidney disease (CKD) to the severity and outcome of COVID-19 patients [[1], [2], [3]]. Given the importance of the basic metabolic panel for timely care in emergent situations it is beneficial to understand patient risk factors and chronic conditions at triage, as well as monitoring of those parameters during treatment and recovery. Although it does not provide a test for directly detecting COVID-19, the new GEM Premier ChemSTAT point-of-care system can provide rapid test results in about a minute for aid in the diagnosis of a patient's acid/base status, electrolyte and metabolite balance.

The new cartridge-based GEM Premier ChemSTAT system was developed with an expanded whole blood testing panel for creatinine and eGFR, BUN, and measured HCO_3_^−^.

In this study, analytical performance of the GEM Premier ChemSTAT was evaluated by non-laboratorian staff at a single center, the Clinical Laboratories of Vall d’Hebron Hospital Campus in Barcelona, Spain for potential use in the POC. This evaluation focused on method comparison to established comparative methods and assessment of within-sample repeatability and long term within-laboratory precision.

## 2 Material and methods

### 2.1 Patient samples

Deidentified, remnant heparinized whole blood specimens were collected at Hospital Vall d’Hebron (Barcelona, Spain). The evaluation protocol was reviewed and approved by the local Institutional Review Board in accordance with the Declaration of Helsinki. Individual participant consent was not required because the study used deidentified remnant blood samples.

### 2.2 Measurement methodologies

#### 2.2.1 GEM Premier ChemSTAT and 4000 systems

The GEM Premier ChemSTAT (hereafter ChemSTAT) (Instrumentation Laboratory, Bedford, MA, US) is a new cartridge based POC testing system that has two primary components: the GEM Premier ChemSTAT instrument and a disposable, all-in-one, multi-use cartridge (GEM PAK) that is stored at room temperature and contains all the materials required to perform analytical testing, including sensors, solutions, sampler, tubing and waste bag.

The ChemSTAT system provides quantitative measurement of Na^+^, K^+^, Ca^++^, Cl^−^, glucose, creatinine, BUN (or urea), tCO_2_, hematocrit, lactate, pH, and pCO_2_ from a single whole blood specimen. The system uses patented Intelligent Quality Management (iQM) technology. Once the GEM PAK is validated, it uses an active quality process control program (iQM) designed to provide continuous monitoring of the analytical process with automatic error detection, attempts to correct any errors, and documentation of all corrective actions, replacing the use of external quality control. This simplifies use in emergency and other POC critical care settings by non-laboratorians. The creatinine assay on ChemSTAT relies on a differential measurement of both creatinine and creatine electrodes. The creatinine/creatine electrodes are amperometric biosensors involving a three/two-enzyme cascading reaction converting creatinine/creatine to hydrogen peroxide (H_2_O_2_). The BUN (urea) assay utilizes a potentiometric sensor consisting of urease enzyme. The bicarbonate sensor relies on a pH selective polymer as a gas permeable outer membrane and an internal bicarbonate buffer. The Na^+^, K^+^, Ca^++^, Cl^−^, pH and pCO_2_ assays are based on a potentiometric ion selective sensor, while the glucose and lactate assays are based on an amperometric biosensor, and the hematocrit assay is based on a conductivity sensor. The working principles of the assays were described in detail in literature [4].

GEM Premier 4000 applies the same measurement technology as the ChemSTAT for all the analytes used as comparative methods in this study.

#### 2.2.2 Beckman AU5800 chemistry analyzer

The reference clinical biochemistry analyzer, Beckman AU5800 (Beckman Coulter Life Sciences, Brea, CA, US) uses an indirect potentiometric ion selective electrode method for Na^+^, K^+^ and Cl^−^ electrolyte measurements. Glucose and urea measurements were both colorimetric enzymatic assays. The creatinine measurement was the kinetic colorimetric assay based on the Jaffe method.

### 2.3 Method comparison study

The method comparison study was performed in accordance with Clinical and Laboratory Standards Institute (CLSI) EP09c [5] with the aim to evaluate the analytical characteristics of the ChemSTAT. Over a period of 8 weeks, 128 heparinized human venous whole blood (WB) samples obtained from the core lab were collected in this study and they came mainly from oncology and nephrology departments. The age range was from 9 to 87 years, with 52 females (41%). A paired draw of a serum (S) sample for basic metabolic profile testing on Beckman AU, and a WB sample in a heparinized syringe for blood gas analysis on GEM 4000 was collected at the same time. Both paired draw samples were received by lab within 30 min. The serum samples were tested on AU5800 first and the WB blood samples were selected for this study after reviewing the serum results on the laboratory information system to optimize the sample range for the method comparison study. Following the routine analysis on GEM 4000, the remnant WB samples were analyzed on ChemSTAT by non-laboratorian staff within 5 min. From the same WB, the separated plasma (P) samples were analyzed on AU5800 within 30 min from ChemSTAT analysis. All samples were kept at room temperature until analysis within 60 min.

Method comparison studies were performed against the AU5800 for creatinine, BUN, Na^+^, K^+^, Cl^−^, and glucose, and also compared against a whole blood POC system, GEM Premier 4000 for Na^+^, K^+^, Cl^−^, Ca^++^, glucose, lactate, hematocrit, pH and pCO_2_.

Five ChemSTAT PAKS were used in this study covering two different sensor and reagent lots. All results except those from quality control material were obtained from a single reagent lot for AU5800 and GEM Premier 4000 systems. Reagents, calibration verification product and QC materials were supplied by the manufacturers.

### 2.4 Precision study

The precision characteristics of ChemSTAT were evaluated according to CLSI EP15-A2 [6] over a period of four months. To estimate the within-laboratory precision (total precision) of the ChemSTAT, three levels of aqueous QC material were used (ChemSTAT System Evaluator Level 1, 2 and 3 and ChemSTAT Hematocrit Evaluator Level 1, 2 and 3, [Instrumentation Laboratory, Bedford, MA, USA]). Testing was performed for all 3 levels to estimate the within lab total repeatability prior to beginning the patient sample testing.

The within-run repeatability was estimated with a whole blood precision study. Heparinized whole blood specimens were assayed in duplicate on one ChemSTAT system to estimate repeatability with total of thirty-four whole blood specimens.

## 3 Statistical analysis

Analyse-it for Microsoft Excel Method Validation edition (version 5.66), an add-in statistical analysis software for MS Excel (Analyse-it Software, Ltd, Leeds, UK) was applied in data analysis and graphic presentation of study results.

Deming (for Na^+^, hematocrit, and pH) or Passing-Bablok (for K^+^, Ca^++^, Cl^−^, glucose, pCO_2_, lactate, creatinine and urea) regression methods were used to obtain slope, intercept, correlation coefficients and confidence intervals. Deming regression was used for analytes with constant variability (SD) and Passing-Bablok regression was used for analytes with mixed variability (SD and coefficient of variation, CV) throughout the measuring range. The bias at the medical decision levels (MDL) for the ChemSTAT versus the AU5800 or GEM Premier 4000 was estimated for each evaluated analyte. The estimated mean bias across tested sample ranges for each analyte was obtained from the Bland-Altman analysis to assess the agreement between ChemSTAT and the comparative methods.

The ChemSTAT analytical performance was deemed acceptable versus the comparative method if the analyte regression slope was within the range of 0.90–1.10 and the correlation coefficient r ≥ 0.95. Biases estimated from regression equation at each Medical Decision Level (MDL) per analyte and should be within the Total Error Allowed (TEa). Bland-Altman analysis was performed to evaluate the agreement of the ChemSTAT test results with the comparative methods by evaluating the mean bias, SD of bias and limits of agreement for 95% sample (LoA). The mean bias should be within TEa and the LoA shall be within the range of clinically acceptable limits. For some evaluated analytes, the regression analysis results were not representative due to a narrow range of samples spanning the claimed measuring range. In those cases, the mean bias from Bland-Altman analysis was used to assess the agreement between two methods and the percentage of samples within the total allowable error (TEa) limit was calculated and compared against the proposed acceptance criterion of ≥95%.

For data analysis of the aqueous control precision study**,** within-laboratory (total) mean, standard deviation (SD), and coefficient of variance (%CV) were computed for each analyte of ChemSTAT System Evaluator Levels 1–3 and ChemSTAT Hematocrit Evaluator Levels 1–3, separately. The study results were deemed acceptable if the estimated within-laboratory (total precision) SD or %CV were less than or equal to the acceptance criteria set by the Spanish Society of Laboratory Medicine (SEQCML) [7,8] and the manufacturer [9].

For the whole blood repeatability study, the coefficient of variation for each sample was computed and each of these %CV was pooled to determine the within-sample %CV and SD for establishing acceptability. The study results were deemed acceptable if the estimated within-sample (within-run repeatability) SD or %CV were less than or equal to the acceptance criteria set by SEQCML and the manufacturer.

## 4 Results

### 4.1 Method comparison study

The regression analysis, using either Deming or Passing-Bablok regression methods, was performed for each evaluated analyte *vs.* AU5800 or GEM Premier 4000. The number of samples, tested sample range, regression slope (95% CI), correlation coefficient (r), and estimated bias at MDL are summarized in Table 1 and Table 2 for ChemSTAT WB vs. AU5800 P or GEM Premier 4000 WB, respectively. Regression slopes ranged from 0.957 to 1.159 vs. AU5800 for creatinine, urea, Na^+^, K^+^, Cl^−^ and glucose and 0.965 to 1.124 vs. GEM Premier 4000 for electrolytes, glucose, lactate, hematocrit, pH and pCO_2_. Intercepts ranged from −1.357 to 2.31 vs. AU5800 and -2.936 to 5.973 vs. GEM Premier 4000. Correlation coefficients (r) were 0.952–0.998 and 0.949 to 0.999 vs. AU5800 and GEM Premier 4000 respectively, for all evaluated analytes. The estimated mean bias between ChemSTAT and the comparative methods for all analytes from the Bland-Altman analysis was within TEa and in the range from −0.21 mmol/L for sodium to +1.34 mg/dL for glucose and from +0.004 for pH to +3.4 mg/dL for glucose vs. AU5800 (Table 1) and GEM Premier 4000, respectively (Table 2).

**Table 1:** Summary of method comparison between GEM Premier ChemSTAT WB and Beckman AU 5800 plasma samples.

Analyte	Sample Count	Min	Max	r	Slope (95% CI)	Mean Bias (SD)	95% CI for Mean Bias	MDL	Bias at MDL (95% CI)	Acceptable criteria (TEa)
**Creatinine (mg/dL)**	125	0.37	10.15	0.993	1.014 (0.9913–1.043)	0.044 (0.263)	−0.0027 - 0.0903	0.60	−0.001 (−0.018 - 0.020)	±0.300
								1.60	0.013 (−0.005 - 0.045)	±0.300
								6.00	1.25% (−1.0% - 4.6%)	±15.0%
**Urea (mmol/L)**	125	2.73	42.37	0.995	0.957 (0.9345–0.9721)	0.02 (0.95)	−0.151 - 0.187	2.1	0.59 (0.42–0.72)	±0.71
								9.3	2.7% (1.1%–3.8%)	±9.0%
								17.9	−0.9% (−2.3% - 0.6%)	±9.0%
**Na**^**+**^**(mmol/L)**	128	124	176	0.960	0.985 (0.9317–1.038)	−0.2 (1.57)	−0.48 - 0.07	115	0.14 (−1.10 - 1.39)	±4.0
								135	−0.15 (−0.47 - 0.16)	±4.0
								150	−0.38 (−1.08 - 0.33)	±4.0
**K**^**+**^**(mmol/L)**	128	2.8	6.6	0.978	1.159 (1.118–1.200)	0.16 (0.16)	0.13 - 0.19	3.0	−0.024 (−0.064 - 0.025)	±0.50
								5.8	0.42 (0.328–0.511)	±0.50
								7.5	9.2% (7.0%–11.4%)	±7.0%
**Cl**^**−**^**(mmol/L)**	124	78	135	0.952	0.988 (0.9118–1.011)	1.1 (2.29)	0.68 - 1.49	90	1.3% (0.9%–2.4%)	±5.0%
								112	0.8% (0.1%–1.4%)	±5.0%
**Glucose (mg/dL)**	124	45	616	0.998	1.024 (1.000–1.047)	1.3 (5.25)	0.41 - 2.28	45	−0.3 (−1.61 - 2.00)	±6.00
								120	1.25% (0.6%–2.0%)	±10.0%
								180	1.63% (0.6%–2.9%)	±10.0%
								350	2.0% (0.3%–3.8%)	±10.0%

MDL - Medical Decision Level, CI – confidence interval, TEa – Total error allowable.

**Table 2:** Summary of method comparison of WB samples between GEM Premier ChemSTAT and GEM Premier 4000.

Analyte	Sample Count	Min	Max	r	Slope (95% CI)	Mean Bias (SD)	95% CI for Mean Bias	MDL	Bias at MDL (95% CI)	Acceptable criteria (TEa)
**Na**^**+**^**(mmol/L)**	128	123	176	0.983	0.965 (0.937–0.992)	1.1 (1.1)	0.93–1.3	115	1.9 (1.2–2.6)	±4.0
								135	1.2 (1.0–1.4)	±4.0
								150	0.7 (0.3–1.1)	±4.0
**K**^**+**^**(mmol/L)**	128	2.6	6.7	0.995	1.000 (1.000–1.056)	0.17 (0.079)	0.1508–0.178	3.0	0.2 (0.10–0.20)	±0.50
								5.8	0.2 (0.10–0.27)	±0.50
								7.5	2.7% (1.3%–4.8%)	±7.0%
**Ca**^**++**^**(mmol/L)**	128	0.93	1.76	0.986	1.056 (1.003–1.093)	0.043 (0.022)	0.039–0.047	0.37	−0.0055 (−0.037 - 0.039)	±0.10
								0.82	0.020 (0.0041–0.040)	±0.10
								1.58	3.9% (2.8%–4.8%)	±10.0%
**Cl**^**−**^**(mmol/L)**	128	80	135	0.974	1.000 (1.000–1.000)	0.77 (1.66)	0.479–1.06	90	1.11% (1.1%–1.1%)	±5.0%
								112	0.89% (0.89%–0.89%)	±5.0%
**Glucose (mg/dL)**	128	42	633	0.999	1.037 (1.025–1.051)	3.4 (3.4)	2.81–3.99	45	1.1 (0.4–1.8)	±6.00
								120	3.20% (2.6%–3.9%)	±10.0%
								180	3.36% (2.5%–4.5%)	±10.0%
								350	3.5% (2.4%–5.0%)	±10.0%
**Lactate (mmol/L)**	128	1.0	5.5	0.994	1.056 (1.000–1.077)	0.05 (0.13)	0.023–0.068	2.0	0.0 (−0.02 - 0.01)	±0.40
								5.0	3.33% (0.0%–4.3%)	±15.0%
**Hematocrit (%)**	122	25	67	0.949	1.124 (1.041–1.207)	1.8% (2.8)	1.28–2.3	21	−0.3 (−1.8 - 1.1)	±4.0
								33	1.1 (0.5–1.8)	±4.0
								56	4.0 (2.4–5.6)	±4.0
pH	128	7.08	7.49	0.988	1.031 (0.996–1.067)	0.004 (0.013)	0.0021–0.0065	7.30	0.004 (0.002–0.006)	±0.04
								7.35	0.006 (0.003–0.008)	±0.04
								7.45	0.007 (0.003–0.011)	±0.04
***p*CO**_**2**_**(mmHg)**	128	29	84	0.993	1.000 (0.967–1.000)	0.1 (1.3)	−0.14 – 0.33	35	0.0 (0.0–0.5)	±5.0
								50	0.0 (0.0–0.0)	±5.0
								70	0% (−0.8% - 0.0%)	±8.0%

MDL - Medical Decision Level, CI – confidence interval, TEa – Total error allowable.

**Fig. 1:**
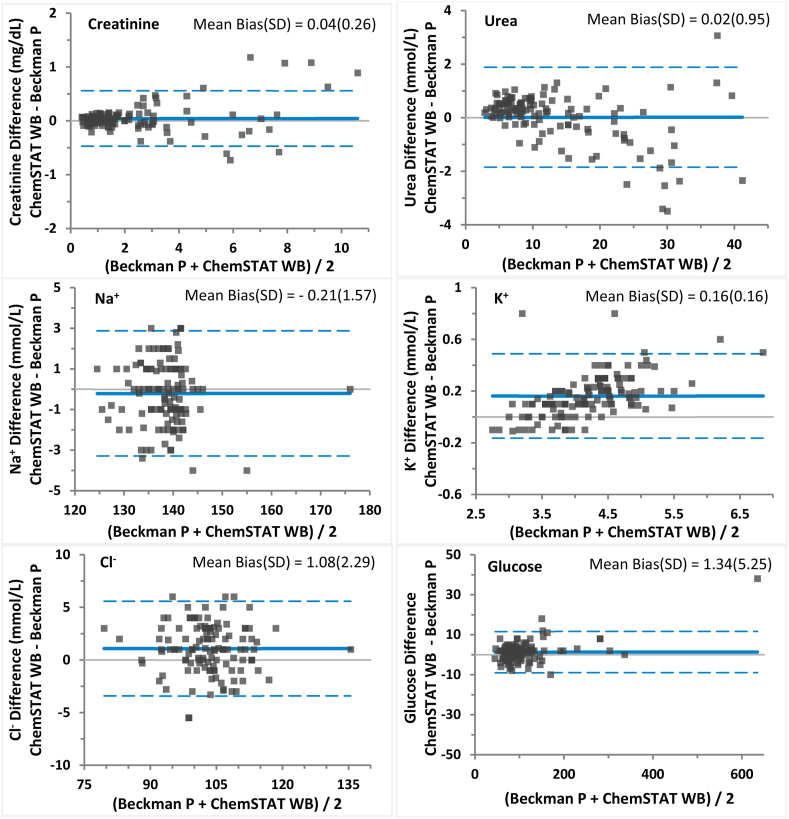
Method Comparison of GEM Premier ChemSTAT whole blood (WB) vs. plasma (P) on Beckman AU5800 analyzer. Bland-Altman chart of absolute difference, average bias (solid line) and 95% LoA (limits of agreement, dashed lines) of creatinine, urea, Na^+^, K^+^, Cl^−^ and glucose vs. AU5800.

**Fig. 2:**
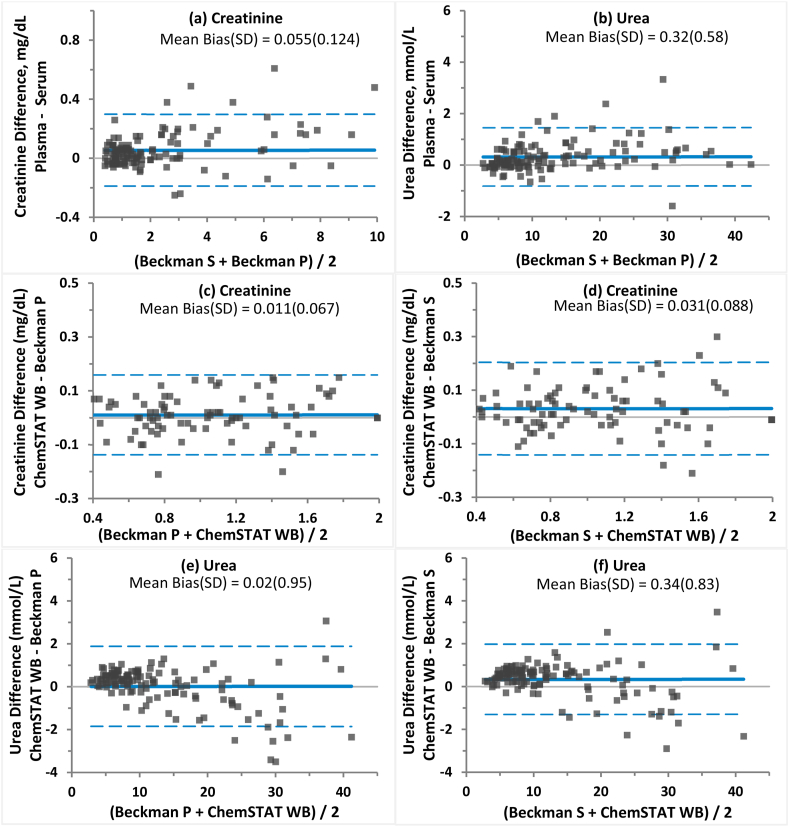
Comparison of Beckman AU5800 creatinine and urea assays in plasma (P) and serum (S) and its impact on GEM Premier ChemSTAT performance. Bland-Altman chart of absolute difference, average bias (solid line) and 95% LoA (dashed lines) of AU5800 creatinine and urea in plasma vs. in serum (a, b). Bland-Altman charts of absolute difference, average bias (solid line) and 95% LoA (dashed lines) of GEM Premier ChemSTAT creatinine (c, d) and urea (e, f) assays vs. Beckman AU5800 plasma or serum results.

For the method comparison analysis *vs.* AU5800 P for creatinine, BUN, Na^+^, K^+^, Cl^−^ and glucose, the regression slopes were all within the recommended range of 0.90–1.10 except for K^+^ which was 1.159 and the correlation coefficients (r) for all the analytes met the recommended criteria of ≥0.95. The slightly elevated regression slope for K^+^ was likely due to difference between the two assays; consistent K^+^ results were observed between whole blood assays on ChemSTAT and GEM Premier 4000 which were assayed within 5 min. Despite the elevated slope, Bland-Altman analysis demonstrated good agreement between ChemSTAT and AU5800 K^+^ results as 98% of the sample biases were within the TEa limit. The method comparison performance for each analyte vs. AU5800 is shown in Fig. 1. The biases estimated at MDLs were all within the TEa and very small for all analytes (<5%) except for K^+^ for the likely cause discussed above. Good agreement between ChemSTAT WB and AU5800 plasma creatinine was observed; biases at MDL1 (0.6 mg/dL) and MDL2 (1.6 mg/dL) were only −0.001 (0.17%) and 0.013 (0.8%), respectively. Fig. 2(c) presents a Bland-Altman bias chart for creatinine up to 2.0 mg/dL (covered 66% of the tested samples) of creatinine for better assessment of scattering at low to normal creatinine sample ranges (mean bias 0.011, SD 0.067).

For the method comparison analysis *vs.* GEM Premier 4000 WB for Na^+^, K^+^, Cl^−^, glucose, lactate, hematocrit, pH, and pCO_2_, the regression slopes were all within the recommended range of 0.90–1.10 except for hematocrit which had a slightly elevated slope of 1.124 and the correlation coefficients (r) for all the analytes met the recommended criteria of higher than 0.95. In this study, the reference hematocrit on GEM Premier 4000 was derived from the measured total hemoglobin by CO-Oximetry while the ChemSTAT hematocrit was measured based on a conductivity method. Difference in measurement methodology and sample inhomogeneity may have contributed to the small deviation of slope from the unity line.

During the study, serum samples were also tested on AU5800 for creatinine and urea in parallel and the results were analyzed to compare with those results from plasma for every patient sample to assess variability between the two matrices on a laboratory chemistry analyzer. For AU5800 Crea and Urea assays, small but noticeable positive biases between plasma and serum samples were observed as shown in Fig. 2 (a, b). Therefore, when comparing the whole blood ChemSTAT assays with different matrix up to 3% discrepancy could occur depending on the sample matrix in the bias analysis as shown in Fig. 2(c–f).

### 4.2 Precision study

For the ChemSTAT, the within-laboratory precision or total precision (SD or CV%) for all the measured parameters were within the specified acceptance criteria in a three level quality control material over the 41 test days. The estimated SD or CV% (highlighted in bold) were compared with the SEQCML criteria and the manufacturer's claim as summarized in Table 3 and both criteria were met for all the analytes. Except for BUN and high level pCO2, all other parameters had an estimated total precision (SD or CV%) less than 50% of the manufacturer's acceptance criteria.

**Table 3:** ChemSTAT total precision study results in external aqueous controls (3-level ChemSTAT System Evaluator [CSE] or 3-level ChemSTAT Hematocrit Evaluator [CHE]).

Analyte	Mean	SD	CV%	Manufacturer specified acceptance criteria (1/2 TEa)
**Creatinine (mg/dL)**	0.85	**0.05**	5.86	0.15 (SD)
	2.48	**0.08**	3.13	0.15 (SD)
	4.67	0.11	**2.33**	7.5% (CV)
**BUN (mg/dL)**	15.6	**0.4**	2.6	1.0 (SD)
	37.5	1.28	**3.4**	4.5% (CV)
	56.9	2.23	**3.92**	4.5% (CV)
**tCO2 (mmol/L)**	25.1	0.64	**2.55**	5% (CV)
	18.8	**0.23**	1.2	1.0 (SD)
	12.8	**0.52**	4.11	1.0 (SD)
**Na**^**+**^**(mmol/L)**	124.1	**0.72**	0.58	2 (SD)
	138.8	**0.71**	0.51	2 (SD)
	151.7	**1.04**	0.68	2 (SD)
**K**^**+**^**(mmol/L)**	2.6	**0.04**	1.69	0.25 (SD)
	4.6	**0.05**	1.17	0.25 (SD)
	7.4	0.07	**0.96**	3.5% (CV)
**Ca**^**++**^**(mmol/L)**	0.9	**0.01**	1.58	0.05 (SD)
	1.1	0.01	**1.36**	5% (CV)
	1.6	0.02	**1.48**	5% (CV)
**Cl**^**−**^**(mmol/L)**	92.2	0.83	**0.9**	2.5% (CV)
	107.1	0.78	**0.73**	2.5% (CV)
	135.6	1.07	**0.79**	2.5% (CV)
**Glucose (mg/dL)**	388.9	3.19	**0.82**	5% (CV)
	115.8	1.39	**1.2**	5% (CV)
	79.2	1.64	**2.06**	5% (CV)
**Lactate (mmol/L)**	8.4	0.2	**2.33**	7.5% (CV)
	5.1	0.18	**3.46**	7.5% (CV)
	1.7	**0.08**	4.92	0.2 (SD)
pH	7.13	**0.008**	0.11	0.02 (SD)
	7.36	**0.004**	0.06	0.02 (SD)
	7.51	**0.005**	0.33	0.02 (SD)
***p*CO**_**2**_**(mmHg)**	90.2	2.78	**3.09**	4% (CV)
	37.4	**0.77**	2.07	2.5 (SD)
	15.7	**0.62**	3.94	2.5 (SD)
**Hematocrit (%)**	22	**0.55**	2.49	2 (SD)
	42	**0.16**	0.37	2 (SD)
	67	**0.38**	0.57	2 (SD)

The within laboratory repeatability study were performed in single run per day on 41 days (total N = 41 per level) over a period of 3 months with 5 GEM PAKs. The pooled SD and CV% are summarized per level. The bold value (either SD or CV%) was used to compare with the manufacturer specified acceptance criteria at the QC level. CV% was used to compare with the SEQC^ML^ (the Spanish Society of Laboratory Medicine) criteria.

The entire data set was also divided into 20, 30 and 40 three test day groups and the mean for the QC materials was estimated for each group. An analysis of variance (ANOVA) was performed to evaluate significant differences between the three models. The results showed no statistically significant difference (p > 0.05) among three groups for all analytes and all 3 tested levels except for tCO_2_ at level 1 (p < 0.05). However, the differences of the means among three groups were within ±0.5 mmol/L (4%) at mean recovery of 12.8 mmol/L; therefore, no clinically significant impact is expected.

The repeatability or the within-run sample precision study were carried out for seventeen test days with a total of 68 whole blood samples by multiple medical staff. Sample results were analyzed and the pooled within-sample precision for all analytes are summarized in Table 4. In the table, the precision results (highlighted in bold) were presented based on either constant SD or %CV was used for assessing the acceptability. The estimated pooled within-sample precision was 0.14%–3.14% for all analytes and the results were all within 50% of the specification for all analytes. An excellent precision (SD of 0.038 or 1.77 CV%) was observed for creatinine across the sample range (0.48–8.13 mg/dL).

**Table 4:** Analysis results of ChemSTAT whole blood precision.

Analyte	Mean	Min	Max	Mean Bias (Rep2-Rep1)	SD of Bias	CV%	Manufacture specified acceptance criteria
pH	7.31	7.08	7.57	−0.004	**0.01**	0.13	0.02 (SD)
***p*CO**_**2**_**(mmHg)**	50.2	34	80	0.2	**1.02**	2.04	2.5 (SD)
**Na**^**+**^**(mmol/L)**	137.4	124	145	0.6	**0.75**	0.55	2 (SD)
**K**^**+**^**(mmol/L)**	4.30	2.7	6.5	0.02	**0.04**	1.02	0.25 (SD)
**Cl**^**−**^**(mmol/L)**	104.5	88	117	−0.6	0.84	**0.80**	2.5% (CV%)
**Ca**^**++**^**(mmol/L)**	1.26	0.95	1.48	0.018	0.018	**1.43**	5% (CV%)
**Glucose (mg/dL)**	120.1	58	325	−1.2	1.38	**1.15**	5% (CV%)
**Lactate (mmol/L)**	2.55	0.8	5.9	0.1	**0.08**	3.18	0.20 (SD)
**Hematocrit (%)**	37.2	17	57	−0.11	**0.92**	2.4	2 (SD)
**BUN (mg/dL)**	37.22	11.7	112	0.26	0.69	**1.86**	4.5% (CV%)
***t*CO**_**2**_**(mmol/L)**	21.22	10.9	30.9	−0.19	0.25	**1.16**	5% (CV%)
**Creatinine (mg/dL)**	2.14	0.48	8.13	−0.02	0.038	**1.77**	7.5% (CV%)

34 whole blood samples were analyzed in duplicates (total replicates N = 68) on ChemSTAT and the pooled within-run imprecision were assessed for each analyte. The bold value (either SD or CV%) was used to compare with the manufacturer specified acceptance criteria.

## 5 Discussion

There has been a published comparison study for ChemSTAT vs. lab analyzer Roche cobas (4) but this study provided unique information in the following aspects: 1) due to the lack of harmonization and commutable reference standards for certain clinical assays, subtle biases exist among assays from different analyzer manufacturers and/or methodology (e.g. Crea, tCO_2_, BUN, and Na^+^). Before adapting a new POC analyzer such as ChemSTAT it is important to evaluate its analytical performance against the institute's existing test method. Comparison of ChemSTAT against Beckman AU5800 was not available in literature. Many hospitals conduct multiple tests for the same patient on both lab and POC analyzers throughout the patient stay for diagnosis and monitoring purposes. Commutability between POC WB assay and lab results is critical for hospitals in deciding which assay to use depending on the urgency of needing assay results. 2) This study evaluated the ChemSTAT creatinine assay against the Jaffe creatinine assay which is widely used on clinical chemistry analyzers while the previous study compared ChemSTAT against the enzymatic creatinine assay. 3) The current study focused on the patients in nephrology and oncology while the previous study evaluated with ED patients. Both studies provided unique clinical performance of the ChemSTAT to specific patient populations.

In this study all assays for whole blood from the ChemSTAT correlated well with plasma samples on the Beckman AU 5800 across the tested sample ranges. The study confirmed equivalent performance of the ChemSTAT system to an established clinical laboratory analyzer for these assays. Although analyzed from whole blood specimens, the accuracy and precision of these assays were consistent throughout the entire sample range. The ChemSTAT WB glucose recoveries agreed well with plasma results on AU5800. This is not surprising because the outer membrane of the biosensor is impermeable to red cells so the biosensor measures the glucose in the aqueous portion. The repeatability was within the SEQCML and manufacturer specified criteria for all analytes. The whole blood Na^+^, K^+^, Ca^++^, Cl^−^, glucose, hematocrit, pH, pCO_2_, and lactate results also correlated well with whole blood results on the GEM Premier 4000 across the tested sample ranges. As a result, the ChemSTAT can provide lab quality whole blood analysis for quick assessment of electrolyte abnormality and renal function especially for patient triage and monitoring in acute care settings such as in COVID-19.

To identify a patient who developed stage 1 of AKI, a creatinine increase by 0.3 mg/dL within 48 h or 1.5 to 1.9 times increase from baseline within 7 days defined by Kidney Disease: Improving Global Outcomes (KDIGO) can be used as criteria. The ChemSTAT reports eGFR values derived from either Modification of Diet in Renal Disease (MDRD) or Chronic Kidney Disease Epidemiology Collaboration (CKD-EPI) equations based on the IDMS traceable whole blood creatinine assay. The small bias (0.011 mg/dL for creatinine up to 2.0 mg/dL with 95% limits of agreement (LoA) from −0.138 to 0.159 mg/dL) and low imprecision (1.8% CV) observed for creatinine in this study made the system capable to predict the change of 0.3 mg/dL in whole blood creatinine. Based on its accuracy and better precision than 0.1 mg/dL, as recommended by the National Kidney Disease Education Program (NKDEP) [10] the ChemSTAT creatinine sensor can provide a reliable eGFR.

In this study, when plasma vs. serum analysis was compared on AU5800, it was noticed that systematic biases (creatinine bias of 0.055 mg/dL with 95% LoA from −0.189 to 0.298 mg/dL, and urea bias of 0.32 mmol/L with 95% LoA from −0.81 to 1.46 mmol/L) were observed between plasma and serum samples for AU5800 creatinine and urea assays. This bias between serum vs. plasma results has also reflected on the ChemSTAT WB vs. AU5800 S as positive biases and wider LoA when correlating to serum samples (for example creatinine bias of 0.031 mg/dL with 95% LoA from −0.141 to 0.204 mg/dL vs. serum as depicted in Fig. 2 d). Increases in estimated ChemSTAT WB biases were observed for both creatinine and urea when comparing ChemSTAT WB with AU5800 S vs. AU5800 P results (Fig. 2 c-f). The plasma samples were separated from the same whole blood specimen tested on the ChemSTAT and serum was collected in separate tube. Attention should be paid for sample matrix effect specific bias and scattering during the method evaluation due to the tight accuracy requirement for creatinine or other assays.

The 41 day within-laboratory precision study was conducted over a period of four months and utilized five cartridge PAKs. The results of excellent total precision of external quality control material demonstrated the system's capability to maintain its long-term analytical performance consistency through the iQM active quality management program.

The ChemSTAT system was easy to use and did not require any troubleshooting or maintenance. The system continuously monitors the analytical process and performs automatic error detection, correction and documentation of all corrective actions, which makes it a suitable system for use in emergency and critical care settings by non-laboratorians.

## 6 Conclusions

Strong correlations were observed in this study between the ChemSTAT system and the comparative methods with excellent precision in analyzing patient samples. The overall total precision of the ChemSTAT was proven to meet the requirement of clinical laboratory. Implementation of the POC ChemSTAT system for basic metabolic panel as well as pH, pCO_2_ and lactate testing could find improved turnaround time from a single whole blood specimen and could support improved patient triage and management.

## Author statement

All authors participated in concept, design, analysis, writing, reviewing, editing and approving the manuscript. All the authors have accepted responsibility for the entire content of this submitted manuscript and approved submission.

## Declaration of competing interest

AEG, RB, MM, RF and EC declare no potential conflicts of interest concerning the research, authorship, and/or publication of this article. CX declares employment by Werfen.
